# Concomitant Lymphocytic Colitis With Recurrent Clostridium difficile Infection

**DOI:** 10.7759/cureus.51606

**Published:** 2024-01-03

**Authors:** Noor M Suleiman, Maya Baiyasi, Tala Al-Saghir, Bryan Daines, Falgun Patel

**Affiliations:** 1 Internal Medicine, Wayne State University School of Medicine, Detroit, USA; 2 Internal Medicine, Henry Ford Health, Detroit, USA; 3 Dermatology, Beaumont Hospital, Dearborn, USA; 4 Internal Medicine, Beaumont Hospital, Dearborn, USA

**Keywords:** concomitant infection, recurrent clostridium difficile infection, chronic watery diarrhea, lymphocytic colitis, microscopic colitis

## Abstract

Microscopic colitis is a clinicopathological diagnosis that is characterized by chronic microscopic inflammation of the colon and presents with chronic watery diarrhea. There are following two subtypes of microscopic colitis: lymphocytic colitis and collagenous colitis. This is a case of a 70-year-old female with a history of *Clostridium difficile* infections who presented with persistent watery diarrhea and was diagnosed with lymphocytic colitis in the setting of a concomitant *C. difficile *infection. Given her clinical presentation, the patient was initiated on empiric treatment for *C. difficile* infection and showed a lack of clinical improvement with persistent watery diarrhea and elevated white blood cell count. The patient’s symptoms resolved upon the confirmatory diagnosis and treatment of lymphocytic colitis. This study illustrates the importance of assessing for, diagnosing, and treating lymphocytic colitis in patients with chronic non-resolving watery diarrhea, especially in the setting of concomitant or recurrent *C. difficile* infections. Additionally, it emphasizes the need for further characterization of the relationship between *C. difficile* infection and microscopic colitis.

## Introduction

Microscopic colitis (MC) is characterized by chronic microscopic inflammation of the colon that presents with chronic watery diarrhea [1-4]. There are following two subtypes of MC: lymphocytic colitis (LC) and collagenous colitis (CC) [4]. Both subtypes are more prevalent in older female populations [2,4]. MC has been linked to medications such as non-steroidal anti-inflammatory drugs (NSAIDs), proton-pump inhibitors (PPIs), and selective serotonin reuptake inhibitors (SSRIs) [2,3,5]. Furthermore, smoking was found to negatively affect the likelihood of remission [3]. Although MC is a known cause of chronic watery diarrhea, it is often overlooked due to its non-specific presentation [3]. Patients may experience symptoms that are intermittent or chronic in nature. Symptoms may include watery non-bloody diarrhea, fecal urgency, abdominal pain, weight loss, or joint pain [3]. Additionally, in order to diagnose microscopic colitis, pathologic findings and clinical presentation both must align. Pathologic findings must show intraepithelial lymphocytosis and normal epithelial architecture on colonic biopsy [6]. Current literature supports the treatment of microscopic colitis with the use of a budesonide taper for a duration of six to eight weeks [7]. According to the American Gastroenterological Association, current guidelines of medical management recommend budesonide 9 mg daily for eight weeks as the first-line agent for the induction of remission in microscopic colitis [8]. Additionally, there have been suggestions for the use of loperamide, both as an effective standalone treatment and as an adjunctive therapy to budesonide [4,7].

Intriguingly, recent case reports have reported a link between lymphocytic colitis and recurrent *Clostridium difficile* infections [1,9]. There have also been reports of collagenous colitis disease courses complicated by this infection [4]. Furthermore, a recent nationwide case-control study conducted in Sweden by Khalili et al. found that gastrointestinal infections, particularly* C. difficile*, are associated with an increased risk of subsequent development of MC, especially in elderly patients [10]. Although the mechanism of MC and its association with *C. difficile* is not well understood, current studies indicate that this is a particular area of clinical relevance that warrants further exploration.

This study was previously presented as a meeting abstract at the 2023 American College of Physicians (ACP) National Conference on April 29, 2023.

## Case presentation

A 70-year-old female presented to the emergency department with a two-week history of malodorous watery diarrhea. Her past medical history was significant for *C. difficile* infections several years before presentation that resolved following an unknown antibiotic course. She also had depression managed with escitalopram and mirtazapine. She denied any recent use of antibiotics, PPIs, or histamine receptor blockers (H2RA). Her social history was significant for a 15-pack-year history of smoking, an unclear history of domestic abuse, and her current residence in a nursing home. Upon arrival, she was found to be hypotensive with a blood pressure of 83/52 mmHg and hypokalemic. She received a 1 L bolus of 0.9% normal saline intravenously followed by maintenance fluids of 0.9% normal saline at a rate of 125 mL/h. She also received electrolyte replacement to replenish her low potassium. Her complete blood count was within normal limits. In addition, a *C. difficile* glutamate dehydrogenase (GDH) and toxin were ordered.

**Figure 1:**
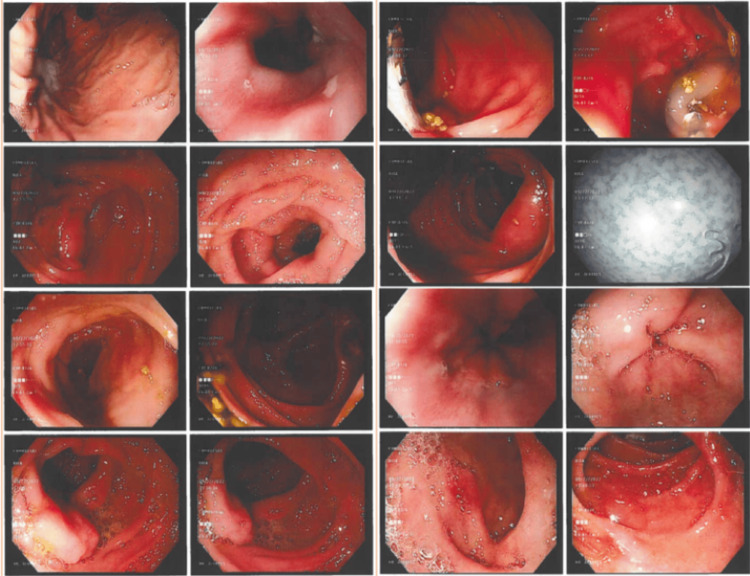
The images during the colonoscopy procedure revealed no macroscopic abnormalities in the colon.

She was later admitted to the general medicine floor for further management. She was placed on maintenance fluids at a rate of 100 mL/h of 5% dextrose in half normal saline due to hypoglycemia with a blood glucose level of 59 units that developed secondary to diarrhea and poor oral intake. She reported arthralgias and myalgias upon arrival to the general medicine floors. Further infectious work-up was ordered which included stool ova and parasite, lactoferrin, and *Cryptosporidium* testing. On day three, stool ova and parasite testing came back negative and ruled out *Giardia lamblia* infection. On day four, she continued to worsen clinically and developed increasing white blood cell count to 13.1 × 10^9^/L. Her lactoferrin results came back positive. On the following day, *C. difficile* GDH and toxin testing came back showing GDH-positive and toxin-negative results. This was suggestive of indeterminate results. Per current infectious disease guidelines, a *C. difficile* nucleic acid amplification test (NAAT) was ordered [11]. Prior to receiving her NAAT results, the decision was made to treat empirically with oral vancomycin due to high suspicion of *C. difficile* infection in the setting of persistent watery diarrhea. In the days that followed, she remained afebrile with persistent diarrhea and worsening leukocytosis. Given her non-resolving diarrhea, gastroenterology was consulted. They recommended holding off on conducting a colonoscopy as they believed her clinical picture was likely due to infection. Infectious disease was consulted and agreed that it was likely due to a non-resolving *C. difficile* infection. They recommended continuing treatment. On day eight, her *C. difficile* NAAT results were positive and leukocytosis began to improve with treatment. In the week that followed, she continued her antibiotic regimen but continued to have persistent non-resolving watery diarrhea. At that point, gastroenterology recommended a colonoscopy and computerized tomography (CT) scan to rule out other etiologies for chronic diarrhea. On day 14, the patient consented to a colonoscopy with random biopsies following a discussion with her primary care physician. She underwent a colonoscopy the following day, which showed no macroscopic abnormalities in the colon (Figure 1).

**Figure 2:**
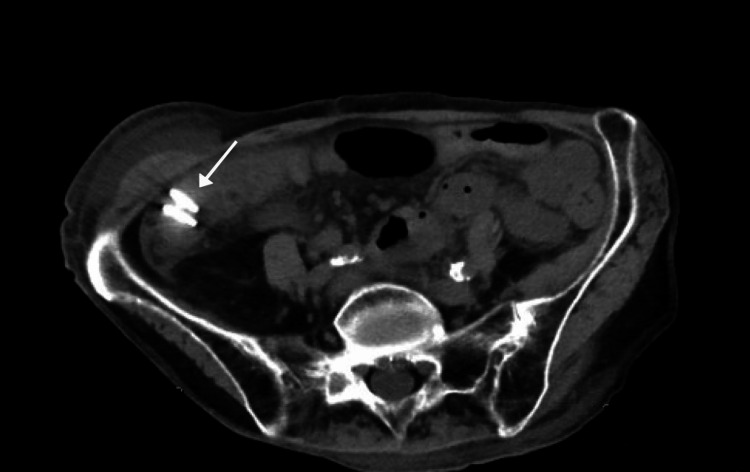
A CT scan of abdomen/pelvis. The white arrow shows the endoclip markers within the right colon and minimal surrounding inflammatory changes, which is indicative of a recent polypectomy.

On day 16, the biopsy results came back showing features that were characteristic of lymphocytic colitis. The pathology report of the biopsy stated that there was increased lamina propria inflammation with patchy mild epithelial lymphocytosis and minimal surface epithelial damage. The CT scan of the abdomen and pelvis showed endoclip markers within the right colon suggestive of a recent polypectomy and minimal surrounding inflammatory changes as indicated by the arrow in Figure 2.

Given the clinical picture and biopsy findings, recommendations were made to begin treatment for lymphocytic colitis. Budesonide at a dose of 9 mg once daily and loperamide 2 mg twice daily were initiated immediately following the diagnosis of lymphocytic colitis. The patient showed marked improvement within 48 h of treatment. She exhibited complete resolution of watery diarrhea and was discharged on a budesonide taper with a follow-up appointment scheduled with a gastroenterologist for outpatient management.

## Discussion

This case presentation reinforces the importance of conducting a colonoscopy with random biopsies in patients with non-resolving diarrhea and a history of recurrent or unresolved *C. difficile* infections to evaluate for lymphocytic colitis. By conducting random biopsies during colonoscopy, medical practitioners can directly examine the intestinal mucosa for characteristic histological changes associated with lymphocytic colitis, thereby enabling timely identification and intervention. Importantly, this study suggests that there are potential relationships between microscopic colitis and *C. difficile* infection that resulted in this patient’s clinical picture. Firstly, it could be that microscopic colitis is a complication that developed secondary to *C. difficile* infection. Secondly, it could be that microscopic colitis developed as a result of the medications used to treat *C. difficile* or other present conditions. Lastly, it could be that patients with microscopic colitis are at higher risk of developing *C. difficile* infections. Based on the presentation of this patient, we believe that she developed a *C. difficile* infection in the presence of lymphocytic colitis, which has been supported by recent case reports that suggested a relationship between these two pathologies [1,9].

Our patient had many of the established risk factors for microscopic colitis. She was an elderly female with a 15-pack-year smoking history. She had a history of depression and was being treated with escitalopram, an SSRI. Of note, she also had a complicated social history of domestic abuse. She lived in a nursing home and displayed a lack of trust in medical staff due to unclear reasons. As a result, there were delays in this patient’s care due to hesitancy to undergo procedures without assistance from her primary care physician, whom she trusted. She had no known PPI, H2RA, or NSAID use. Interestingly, Masclee et al. conducted a case-control study that found that NSAIDs and PPIs were associated with increased risk of developing MC, however, other drugs like SSRIs likely increased the risk of diarrhea in the setting of MC but did not contribute to the development of the disease [5]. Given this information, her history of SSRI use may have contributed to her diarrhea but likely did not predispose her to develop this form of microscopic colitis. Other established risk factors for MC include older age, female sex, and smoking. This patient had all of these risk factors. Interestingly, smoking has been associated with persistent disease and was found to contribute to a lower probability of achieving disease remission [2]. Thus, recommending smoking cessation was important in the management of this patient and other patients with her pathology. Given her history of *C. difficile* infection, there is uncertainty regarding the treatments she previously received and how they may have contributed to the development of MC. Of note, some cases have emphasized a potential association between fecal microbiota transplant and the development of MC [1,9]. There was no documentation in her medical charts of any treatments for previously known and documented *C. difficile* infections, so it is unclear whether our case would support that association. Per reporting from our patient, she had only received treatment with an unspecified antibiotic and her infections had responded well to the medication.

Additionally, lymphocytic colitis may have developed in our patient as a complication secondary to recurrent *C. difficile* infection and a concomitant infection. Khalili et al. stated that the prevalence of previous gastrointestinal infections in patients diagnosed with MC was higher than in controls, 7.5% and 3.0%, respectively. As noted previously, they identified *C. difficile* as one of the gastrointestinal pathogens that showed an increased risk of developing MC [10]. It is however important to note that Khalili et al. found a stronger association with the CC subtype (OR: 3.23) than with the LC subtype (OR: 2.51). Previous cases have reported an association between recurrent *C. difficile* and the subsequent development of LC, especially in *C. difficile* infections that were resistant to treatment [1,9]. While it is unclear what our patient developed first, her recurrence of *C. difficile* infections supports the theory that a recurrence of this infection may be a risk factor for the development of lymphocytic colitis. Further investigation into the potential causal relationship through a broader scale study is warranted to assess the necessity of evaluating for lymphocytic colitis in patients with a history of recurrent *C. difficile* infections.

The other potential explanation for this patient’s presentation is that she developed another *C. difficile* infection secondary to lymphocytic colitis. Given that this patient is a poor historian with poor documentation of previous infections in her medical chart, it is unclear whether this patient had undergone treatment for the *C. difficile* infections. There was documentation of two past *C. difficile* infections, one in 2015 and another in 2019. Per the patient's reporting, she received antibiotics, but she could not recall the name of the drug. Given that she developed leukocytosis later in the hospital stay, it is possible that she developed a *C. difficile* infection in the setting of lymphocytic colitis. Following treatment of *C. difficile*, her symptoms persisted and resolved quickly following the diagnosis and treatment of lymphocytic colitis. This brings to question whether she could have developed these *C. difficile* infections secondary to this underlying inflammatory condition. It is possible that the patient’s gut microbiota was negatively affected due to colonic inflammation, which predisposed her to an increased risk of gastrointestinal infection, more specifically *C. difficile*. While this is less supported by current literature, we recommend consideration of the possibility that this patient population is at greater risk of developing *C. difficile *infection. If this is true, it would suggest the need for routine testing in symptomatic patients with microscopic colitis for this concomitant infection. Early detection can lead to prompt treatment, which we speculate would mitigate worse outcomes in this patient population, especially given the recurrent and treatment-resistant nature of MC. We encourage further research into this potential relationship with the assessment of the incidence and prevalence of gastrointestinal infections in this patient population. If a strong association is identified, we recommend further efforts to understand the mechanism behind this association.

## Conclusions

This study report highlights the relationship between lymphocytic colitis and recurrent *C. difficile* infections. The patient’s initial clinical presentation, persistent non-resolving watery diarrhea despite antibiotics, and a history of recurrent *C. difficile* infections suggest an additional layer of complexity to diagnosing patients with chronic diarrhea. The diagnostic process in this patient was further challenged by their initial reluctance to undergo procedures, which contributed to delays in care. Through a comprehensive evaluation that included colonoscopy with random biopsies, a definitive diagnosis of lymphocytic colitis was made resulting in a rapid improvement in symptoms upon treatment. This suggests the necessity for further evaluation of patients with a history of *C. difficile* infections for possible microscopic colitis. Further investigations are warranted to confirm the mechanism of these associations and better characterize risk factors that predispose patients to concurrent infections. Most importantly, this report emphasizes the importance of comprehensive evaluation of patient history, timely diagnostic strategies, and interdisciplinary collaboration in managing complex cases.
